# Case report: Bilateral panuveitis resembling Vogt-Koyanagi-Harada disease after second dose of BNT162b2 mRNA COVID-19 vaccine

**DOI:** 10.3389/fimmu.2022.967972

**Published:** 2022-09-29

**Authors:** Tomohito Sato, Ryotaro Nihei, Daisuke Sora, Yoshiaki Nishio, Masaru Takeuchi

**Affiliations:** Department of Ophthalmology, National Defense Medical College, Saitama, Japan

**Keywords:** cyTOF, heat map, multiplex bead analysis, uveitis, Vogt-Koyanagi-Harada disease, BNT162b2 vaccine, covid-19 mRNA vaccines

## Abstract

Coronavirus disease 2019 (COVID-19) caused by severe acute respiratory syndrome coronavirus 2 (SARS-CoV-2) remains a serious pandemic. COVID-19 vaccination is urgent needed for limiting SARS-CoV-2 outbreaks by herd immunity. Simultaneously, post-marketing surveillance to assess vaccine safety is important, and collection of vaccine-related adverse events has been in progress. Vision-threatening ophthalmic adverse events of COVID-19 vaccines are rare but are a matter of concern. We report a 45-year-old Japanese male with positive for HLA-DR4/HLA-DRB1*0405, who developed bilateral panuveitis resembling Vogt-Koyanagi-Harada (VKH) disease after the second dose of Pfizer-BioNTech COVID-19 mRNA (BNT162b2) vaccine. Glucocorticosteroid (GC) therapy combined with cyclosporine A (CsA) readily improved the panuveitis. The immune profile at the time of onset was analyzed using CyTOF technology, which revealed activations of innate immunity mainly consisting of natural killer cells, and acquired immunity predominantly composed of B cells and CD8^+^ T cells. On the other hand, the immune profile in the remission phase was altered by GC therapy with CsA to a profile composed primarily of CD4^+^ cells, which was considerably similar to that of the healthy control before the vaccination. Our results indicate that BNT162b2 vaccine may trigger an accidental immune cross-reactivity to melanocyte epitopes in the choroid, resulting in the onset of panuveitis resembling VKH disease.

## Introduction

Coronavirus disease 2019 (COVID-19) caused by severe acute respiratory syndrome coronavirus 2 (SARS-CoV-2) is an ongoing pandemic (1). Regarding the epidemiologic characteristics of COVID-19 outbreak between 2019 and 2020, although the infection rate of COVID-19 varies greatly depending on country and region, generally 30% to 50% of COVID-19 patients were asymptomatic, 20% became severe, and 5% required mechanical ventilation management, half of those under ventilation management died (2, 3). As for the immune profiles of severe COVID-19 patients with acute respiratory distress syndrome (ARDS), cytokine release syndrome (CRS) may occur in these patients, which is a life-threatening systemic inflammatory syndrome involving elevated levels of circulating cytokines and immune cell hyperactivation (4). In COVID-19 patients with ARDS, the levels of interleukin (IL)-1, tumor necrosis factor alpha (TNFα) and IL-6 are elevated in the peripheral blood (5, 6). In addition, severe COVID-19 patients display immune dysregulation manifesting macrophage activation as well as depletion of CD4^+^ T cells and natural killer (NK) cells (7).

At present, COVID-19 vaccination is urgently implemented for limiting SARS-CoV-2 transmission, with the aim to reach a state of so-called herd immunity (8). Several types of novel COVID-19 vaccines including a nucleoside-modified messenger ribonucleic acid (mRNA) have been approved (1). Post-marketing surveillance to assess safety of the vaccines is extremely important, and has been performed to collect adverse events related to COVID-19 vaccines (1). From the millions of doses of mRNA COVID-19 vaccines administered, general adverse events that occur in a considerable subset of individuals are mild to moderate reactions at the injection site, fever, fatigue, body aches, and headache (9). Furthermore, rare but serious adverse effects including vasculitis, thrombosis, cerebral infarction, myocardial damage and multiple organ dysfunction have been reported (5, 10). Regarding COVID-19 vaccine-related ophthalmic adverse events, uveitis, acute macular neuroretinopathy, central serous retinopathy, multiple evanescent white dot syndrome, etc. are reported (11). A few case studies on new onset of Vogt-Koyanagi-Harada (VKH) disease after adenovirus vector COVID-19 vaccine (Oxford AstraZeneca; ChAdOx1 nCoV-19/AZD1222) (12) and mRNA COVID-19 vaccine (Pfizer-BioNTech; BNT162b2) (13) have been published, although the onset mechanism of ophthalmic adverse events from the vaccines remains unclear.

Here we report a rare case of bilateral panuveitis resembling VKH disease occurring after the second dose of BNT162b2 vaccine, and present the immune profile of the patient including analysis of serum cytokines and cell-mediated immunity using multiplex bead analysis system and mass cytometry by time-of-flight (CyTOF) technology.

## Case report

A 45-year-old Japanese male with no medical history of COVID-19 received the second dose of BNT162b2 vaccine. One day after the vaccination, headache and general malaise occurred, but disappeared spontaneously. Four days later, he visited a local doctor with complaint of distorted vision and color vision deficiency. The doctor confirmed the onset of serous retinal detachment (SRD) in the macula of the left eye, and he was referred to the ophthalmology department of our hospital for detailed investigations.

**Figure 1:**
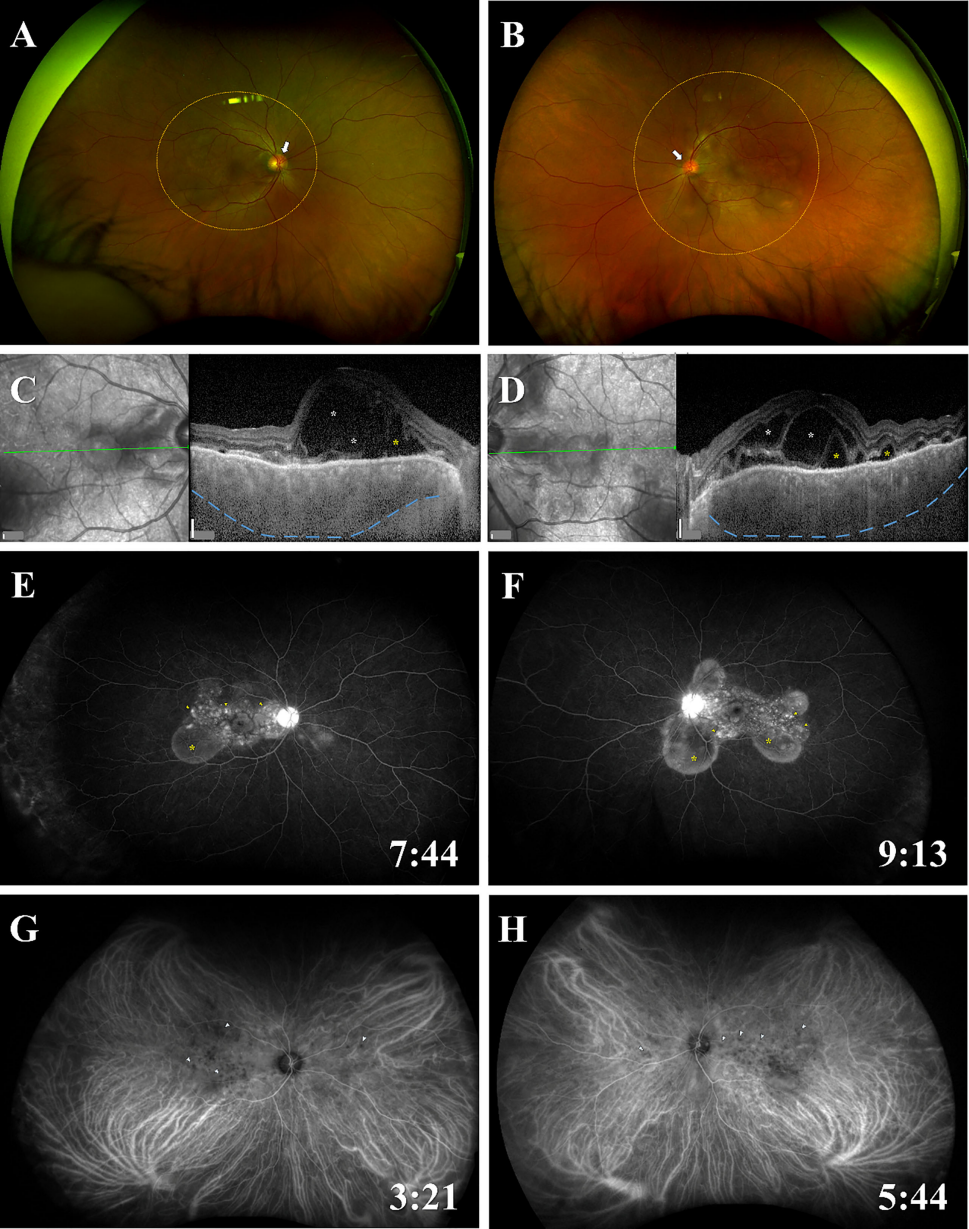
Fundus findings of panuveitis at the time of onset. Color fundus photographs show bullous SRDs in the posterior retina (areas of orange dotted circles), redness and swelling of the optic disc (white arrows) in **(A)** the right eye and **(B)** the left eye. EDI-OCT images reveal SRDs (yellow asterisks), cystoid spaces in the neurosensory layer of the retina (white asterisks), choroidal thickening (blue dotted lines) in **(C)** the right eye and **(D)** the left eye. FA images indicate multiple punctate fluorescein leaks (yellow arrowheads) and pooling (yellow asterisks) consistent with the SRD locations, and hyperfluorescence of the optic disc in **(E)** the right eye and **(F)** the left eye. IA images present dark patches (blue arrowheads) in **(G)** the right eye and **(H)** the left eye. The time of photography after administration of FA or IA is indicated in the lower right corner. Scale bars (white vertical bar) in **(C, D)**, 200 μm. EDI-OCT; enhanced depth imaging optical coherence tomography; FA, fluorescein angiography; IA, indocyanine green angiography; SRD, serous retinal detachment.

At presentation, his best corrected visual acuity (BCVA) on the decimal chart was 0.4 (0.40, when converted to logarithm of the minimum angle of resolution) in the right eye, and 0.6 (0.22) in the left eye. Slit-lamp examination detected grade 1+ inflammatory cells in the anterior chamber of the right eye, and grade 2+ in the left eye, while vitreous opacities was grade 0 in both eyes (14). Color fundus photography showed bullous SRDs in the posterior retina of both eyes (**Figure 1**). SRDs, cystoid spaces in the neurosensory layer of the retina, and choroidal thickening were revealed by enhanced depth imaging optical coherence tomography. Fluorescein angiography (FA) images indicated multiple punctate fluorescein leakages and pooling consistent with the SRD locations, and hyperfluorescence of the optic disc. Indocyanine green angiography (IA) images depicted dark patches, implying inflammatory granuloma in the choroid.

On admission, body temperature was 37.1 degrees Celsius (°C), and there was no abnormal shadow on chest X-ray image. He did not notice tinnitus or onset of hair loss or gray hair. Peripheral blood tests showed white blood cell (WBC) count of 9.90 x 10^3^ cells/μL, and differential counts of neutrophils 67.1%, lymphocytes 28.3% and monocytes 3.3% (**Supplementary Table 1**). Soluble IL-2 receptor level was 476 U/mL and erythrocyte sedimentation rate was 18 mm/hour, whereas C-reactive protein level was 0.3 mg/dL or less. In human leukocyte antigen (HLA) typing, HLA-DR4/HLA-DRB1*0405 and HLA-DR9/HLA-DRB1*0901 were positive. Diagnostic lumbar puncture revealed no pleocytosis (1 cell/mm^3^ in cerebrospinal fluid, **Supplementary Table 2**), implying exclusion of meningitis. Based on the clinical findings and laboratory data, this case was diagnosed as BNT162b2 vaccine-related bilateral panuveitis resembling VKH disease, which is categorized as “probable” based on the presence of ocular and extraocular manifestations (15). As for drug-induced uveitis, we assessed the involvement of BNT162b2 vaccine in the panuveitis by the Naranjo score (16, 17), and judged it to be “probable” with a score of 5.

Glucocorticosteroid (GC) therapy was initiated according to the standard treatment for VKH disease (18). In the clinical course of treatment (**Supplementary Figure 1**), we paid attention to deterioration of visual acuity and subfoveal choroidal thickness, and tapered the dose of prednisolone in accordance with the assessment of uveitis activity proposed by The standardization of Uveitis Nomenclature (SUN) Working Group (14). At six months, subfoveal choroidal thickness in the right and left eyes deteriorated to 553 μm and 607 μm respectively, while visual acuity was well preserved, and the panuveitis was inactive (14) under administration of 12.5 mg/day prednisolone. In addition, the onset of steroid-induced diabetes mellitus was worried because HbA1c level was elevated to 7.1%. Therefore, we determined that combination therapy with prednisolone and cyclosporine A (CsA) was more appropriate than the prednisolone monotherapy to maintain the inactivation of the panuveitis. CsA was started at a dose of 250 mg/day (3.47 mg/kg/day) orally. Approximately seven months later, the multiple SRDs disappeared and BCVA recovered to 1.5 (1.30) in the right eye, and 1.5 (1.30) in the left eye. Fundus findings in the inactive phase are presented in **Supplementary Figure 2**. Results of laboratory tests are described in **Supplementary Table 3**.

To examine the immune profile of this case, peripheral blood samples were collected at the time of onset (Pre-P) and approximately seven months later (Post-P). Control samples from a healthy individual (co-author, male in the thirties) were obtained before BNT162b2 vaccination (Pre-C) and one month after the third BNT162b2 vaccination (Post-C). Serum cytokine levels were investigated by a multiplex bead analysis system (Bio-Plex Human Cytokine 27-plex panel; Bio-Rad, Hercules, CA, USA), and cell-mediated immunity was evaluated by CyTOF technology using the Maxpar Direct ImmunoPhenotyping Assay (MDIPA; Fluidigm, South San Francisco, CA, USA) (19), according to manufacturer’s protocols. Serum levels of platelet derived growth factor-BB, IL-1 receptor antagonist, IL-7, IL-9, eotaxin, interferon-γ-inducible protein-10, macrophage inflammatory protein-1β, regulated on activation, normal T cell expressed and secreted were considerably higher compared with the levels reported for healthy individuals (20) (**Table 1**). On the other hand, serum levels of the remaining cytokines were within normal ranges.

**Table 1:** Serum cytokines levels of the patient with panuveitis at the time of onset.

Cytokine	Level	Detection range	
		Lower	Upper
PDGF-BB	2449.1	*7.67*	*40371*
IL-1β	0.69	*0.28*	*4203*
IL-1ra	35.56	*9.56*	*10863*
IL-2	0	*1.50*	*8778*
IL-4	1.53	*0.16*	*2536*
IL-5	0	*4.02*	*67622*
IL-6	0	*0.37*	*5970*
IL-7	33.67	*10.9*	*36230*
IL-8	1.18	*0.75*	*9710*
IL-9	23.29	*0.92*	*21827*
IL-10	0	*0.84*	*11610*
IL-12	0	*1.85*	*7840*
IL-13	0	*1.41*	*5003*
IL-15	0	*231.8*	*72994*
IL-17A	3.13	*2.69*	*10948*
Eotaxin	107.12	*0.09*	*1487*
bFGF	6.98	*3.52*	*3769*
G-CSF	0	*55.0*	*70103*
GM-CSF	0	*0.42*	*1823*
IFN-γ	0	*0.74*	*10943*
IP-10	1376.26	*18.4*	*23765*
MCP-1	0	*2.61*	*6960*
MIP-1α	0.59	*0.08*	*349*
MIP-1β	37.51	*1.42*	*1481*
RANTES	992.4	*1.44*	*5544*
TNFα	6.38	*2.73*	*53797*
VEGF-A	0	*69.9*	*69174*

Cytokine levels are expressed as pg/mL. Cytokine levels below detectable levels are treated as 0 (21). bFGF, basic fibroblast growth factor; G-CSF, granulocyte colony-stimulating factor; GM-CSF, granulocyte macrophage colony-stimulating factor; IFN-γ, interferon-gamma; IL, interleukin; IL-1ra, IL-1 receptor antagonist; IP-10, interferon gamma-induced protein 10; MCP-1, monocyte chemotactic protein-1; MIP, macrophage inflammatory protein; PDGF, platelet derived growth factor; RANTES, regulated on activation normal T-cell expressed and secreted; TNFα, tumor necrosis factor alpha; VEGF, vascular endothelial growth factor.

**Figure 2:**
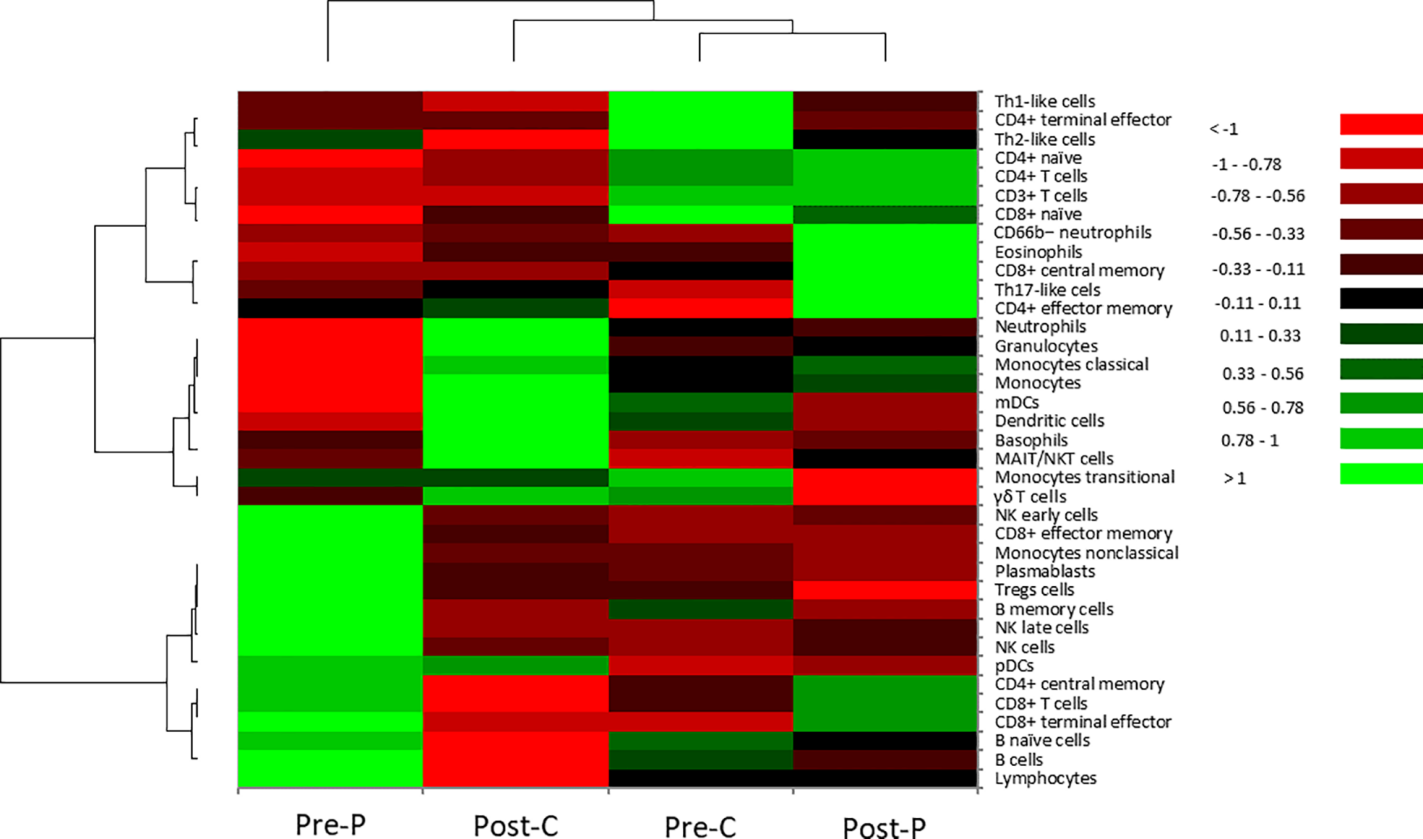
Hierarchical cluster analysis of cellular phenotypes in the patient and the healthy control. In the heatmap, the vertical axis shows 37 types of immune cells in peripheral blood mononuclear cells. The horizonal axis shows four samples: Pre-P; patient not receiving GC therapy at the time of onset, Post-C; healthy control one month after the third dose of BNT162b2 vaccine, Pre-C; healthy control before BNT162b2 vaccination, Post-P; patient receiving GC therapy combined with CsA in the inactive phase. Hierarchical cluster analysis was performed using Euclidean distance as a distance measure and Ward’s method for hierarchical clustering (21). Color scale: low values, red; middle to high values, black to green.

Next, immune cell populations and cellular phenotypes in peripheral blood mononuclear cells were examined (**Table 2** and **Figure 2**). Comparing Pre-P of the patient at the time of onset with Pre-C as BNT162b2 vaccine-naïve control, the cell populations of all CD8^+^ T cells except naïve, CD4^+^ central memory T cells, B cells and NK cells were increased in Pre-P, whereas those of CD8^+^ naïve T cells, CD4^+^ naïve T cells, T helper 1 (Th1)-like cells and T helper 2 (Th2)-like cells were relatively high in Pre-C. The immune profile of Post-P in the inactive phase was substantially similar to that of Pre-C, and the cell populations of CD8^+^ central memory T cells, CD4^+^ effector memory T cells, T helper 17 (Th17)-like cells, CD66b^−^ neutrophils and eosinophils were increased compared to those at the time of onset (Pre-P). When we compared the immune profiles between Pre-C and Post-C to assess the efficacy of BNT162b2 vaccine in the healthy control, the cell populations of neutrophils, basophils, monocytes classical cells, and myeloid dendritic cells (mDCs) were increased in Post-C, while the cell populations of CD8^+^ naïve T cells, CD4^+^ naïve T cells, CD4^+^ terminal effector T cells, Th1-like cells, and Th2-like cells were relatively high in Pre-C. The heat map (**Figure 2**) displaying the distribution of immune cell populations in the four samples indicated that the immune profile of Pre-P was not similar in properties to the profiles of the other three samples (Pre-C, Post-C and Post-P), while there was a high property similarity between the immune profiles of Pre-C and Post-P.

**Table 2:** Immune cell populations and cellular phenotypes in peripheral blood mononuclear cell samples from the patient and healthy control.

Immune cell population					Cellular phenotype		Pre-C	Post-C	Pre-P	Post-P
Intact live cells (%)							100	100	100	100
	Lymphocytes				CD3 T cells + B cells + NK cells + plasmablasts		61.6	41.9	80.7	63.7
		CD3^+^ T cells			CD8 T cells + CD4 T cells + γδ T cells + MAIT/NKT cells		47.4	33.3	33.9	47.4
			CD8^+^ T cells		CD3+ CD66b- CD19- CD8+ CD4- CD14- CD161- TCRγδ- CD123- CD11c-		14.6	11.7	17.7	17.6
				Naïve	CD8 T cells + CD45RA+ CCR7+ CD27+		7.26	4.02	0.88	5.35
				Central memory	CD8 T cells + CD45RA- CCR7+ CD27+		0.13	0.04	0.05	0.31
				Effector memory	CD8 T cells + CCR7- CD27+		1.60	2.26	4.67	1.43
				Terminal effector	CD8 T cells + CCR7- CD27-		5.61	5.39	12.1	10.5
			CD4^+^ T cells		CD66b- CD3+ CD8- CD4+ CD14- TCRγδ- CD11c-		26.2	13.3	11.9	28.0
				Naïve	CD4 T cells + CD45RA+ CCR7+ CD27+		14.9	6.11	3.95	16.5
				Central memory	CD4 T cells + CD45RA- CCR7+ CD27+		2.62	2.00	3.21	3.03
				Effector memory	CD4 T cells + CD45RA- CCR7- CD27+		0.00	4.01	3.48	6.60
				Terminal effector	CD4 T cells + CD45RA- CCR7- CD27-		8.91	1.21	1.22	1.82
				Treg cells	CD4 T cells + CD25+ CD127- CCR4+		0.44	0.43	0.81	0.21
				Th1-like cells	CD4 T cells + CXCR3+ CCR6- CXCR5- CCR4-		1.35	0.22	0.41	0.57
				Th2-like cells	CD4 T cells + CXCR3- CCR6- CXCR5- CCR4+		2.83	1.38	2.35	2.25
				Th17-like cells	CD4 T cells + CXCR3- CCR6+ CXCR5- CCR4+		0.68	1.30	1.06	2.38
			γδ T cells		CD66b- CD3+ CD8dim,- CD4- CD14- TCRγδ dim,+		6.51	7.28	4.19	1.40
			CD4^-^ T Cells						
				MAIT/NKT cells	CD66b- CD3+ CD4- CD14- CD161+ TCRγδ- CD28+ CD16-		0	0.99	0.18	0.45
		B cells			CD3- CD14- CD56- CD16 dim,- CD19+ CD20+ HLA-DR dim,+		11.8	4.95	15.4	9.38
			Naïve		B cells + CD27-		9.37	3.41	11.4	7.90
			Memory		B cells + CD27+		2.38	1.44	3.54	1.46
			Plasmablasts		CD3- CD14- CD16-,dim CD66b- CD20- CD19+ CD56- CD38++ CD27+		0.07	0.09	0.41	0.01
		NK cells			CD14- CD3- CD123- CD66b- CD45RA+ CD56 dim,+		2.43	3.67	31.4	6.92
			Early		NK cells + CD57-		1.52	2.61	11.4	1.83
			Late		NK cells + CD57+		0.91	1.06	20.0	5.09
	Monocytes				CD3- CD19- CD56- CD66b- HLA-DR+ CD11c+		9.65	11.9	6.87	9.88
		Classical			Monocytes + CD14+ CD38+		8.55	11.0	5.09	9.54
		Transitional			Monocytes + CD14 dim CD38 dim		0.82	0.62	0.61	0.20
		Nonclassical			Monocytes + CD14- CD38-		0.27	0.23	1.17	0.14
	Dendritic cells				pDCs+ mDCs		0.48	0.79	0.20	0.24
		Plasmacytoid DCs			CD3- CD19- CD14- CD20- CD66b- HLA-DR dim,+ CD11c- CD123+		0.03	0.11	0.12	0.04
		Myeloid DCs			CD3- CD19- CD14- CD20- HLA-DR dim,+ CD11c dim,+ CD123- CD16 dim,- CD38 dim,+ CD294- HLA-D		0.45	0.68	0.08	0.20
								
	Granulocytes				Neutrophils + basophils + eosinophils + CD66b- neutrophils		17.0	35.9	3.71	18.7
		Neutrophils			CD66b dim,+ CD16+ HLA-DR-		16.4	32.5	2.83	13.0
		Basophils			HLA-DR- CD66b- CD123 dim,+ CD38+ CD294+		0.04	2.37	0.48	0.36
		Eosinophils			CD14- CD3- CD19- HLA-DR- CD294+ CD66b dim,+		0.25	0.27	0.13	0.61
		CD66b^-^ neutrophils			CD3- CD19- CD66b- CD56- HLA-DR- CD123- CD45-		0.32	0.75	0.27	4.74

Cellular phenotypes in this table are as defined by Bagwell et al. (19). Nomenclature such as TCRγδ dim,+ means that dim to positive events are selected. CsA, cyclosporine A; DCs, dendritic cells; GC, glucocorticosteroid; HLA, human leukocyte antigen; MAIT cells, mucosal associated invariant T cells; mDCs, myeloid DCs; NK cells, natural killer cells; NKT cells, natural killer T cells; pDCs, plasmacytoid DCs; Post-C, healthy control one month after the third dose of BNT162b2 vaccine; Post-P, patient receiving GC therapy combined with CsA in the inactive phase; Pre-C, healthy control before BNT162b2 vaccination; Pre-P, patient not receiving GC therapy at the time of onset; Th1-like cells, T helper 1-like cells; Th2-like cells, T helper 2-like cells; Th17-like, T helper 17-like cells.

## Discussion

To the best of our knowledge, we present the first immunological profiling of a patient with BNT162b2 vaccine-related ophthalmic adverse events. Three main conclusions can be drawn (1). In this patient, CRS did not occur, but innate immunity mainly consisting of NK cells, and acquired immunity predominantly composed of B cells and CD8^+^ T cells were activated after the second dose of BNT162b2 vaccine (2). GC therapy combined with CsA suppressed the activated innate and acquired immunities, and changed the immune profile to one predominantly composed of CD4^+^ cells, which had similar properties to the profile of a healthy control before BNT162b2 vaccination (3). After the third dose of BNT162b2 vaccine, the immune profile of the healthy control changed from a profile based on mainly acquired immunity composed predominantly of CD4^+^ T cells to a profile based on innate immunity consisting of monocytes, dendritic cells and granulocytes.

Currently, there are some discussions about the relationship between COVID-19 vaccination and the onset of uveitis with unknown etiology, which is very rare but nonetheless a significant adverse event. Since the innate immune system primed by the first dose of COVID-19 mRNA vaccination elicits a stronger response after the boost dose (22), we sought to examine which immune response triggers the panuveitis resembling VKH disease in this case.

VKH disease is an acute panuveitis characterized by bilateral, diffuse granulomatous lesions resulted from stromal choroiditis, accompanied by neurologic manifestations of headache and nausea (23). Characteristic ocular manifestations of VKH disease at the time of onset include sudden blurred vision, bilateral panuveitis, diffuse granulomatous choroiditis with exudative retinal detachment, vitritis and optic disc swelling (18, 24). The prevalence of VKH disease varies among different countries and ethnic groups (18). In Asian, VKH disease represents one of the most common uveitis entities (25). VKH disease is a complex disease involving multiple interactions among different immune cell populations (18). VKH patients are sensitized to melanocyte epitopes, and patients with HLA-DR4/HLA-DRB1*0405 recognize a broader melanocyte derived peptide repertoire (26). So, HLA-DR4/HLA-DRB1*0405 has been well-known as a significant genetic risk factor involved in the onset of VKH disease (27).

Accumulating evidence indicates that dysregulated T cell subsets including cytotoxic T cells (28), Th1 cells (29), and Th17 cells (30, 31) are an immunological feature in the pathology of VKH disease. Furthermore, activated T cells are demonstrated as the predominant cell types in choroidal inflammation, accompanied by the presence of choroidal melanocytes expressing HLA-DR (32). Our patient is HLA-DR4/HLA-DRB1*0405–positive, and the increase in immune cell population of CD8^+^ T cells would be almost synonymous with the activation of T cell subsets in the interpretation of immune responses. In addition, the increased cell populations of B cells and NK cells at the time of onset in this case were noteworthy findings, and they were similar to the immunological features of VKH disease. B cells contribute to the pathogenesis of autoimmune diseases by producing autoantibodies and/or through acting as antigen-presenting cells (APCs) (33). In VKH patients, B cell infiltration was found in uveal tissues (34). It is presumed that NK cells limit autoimmune responses by inhibiting the proliferation and activation of auto-reactive T lymphocytes, and hampering the activation of monocytes (18). Therefore, the increased NK cells observed in our case may play a counterbalance role to suppress excessive activations of B cell subsets and CD8^+^ T cells for maintaining immune homeostasis.

Although numerous new drugs have been developed within the last few decades, high-dose GC pulse therapy has been the most widely used treatment for VKH disease at the time of onset, because most patients respond well, and achieve symptom amelioration within a few days of the therapy (35). GC therapy has a direct or indirect inhibitory effect on T cells through inhibition of T cell activation, suppression of inflammatory mediators such as nitric oxide, and promotion of apoptosis in immune cells (36). Regarding fluctuation of immune profile in VKH disease by GC therapy, GC therapy not only inhibits T cell activation directly, but also affects monocyte subsets, which might synergistically inhibit the pathogenic immune responses in VKH disease (18). In addition, GC therapy is also effective for the treatment of autoimmune diseases such as rheumatoid arthritis, in which autoantibodies contribute to the pathology (37). On the other hand, CsA is an antibiotic, lipophilic cyclic polypeptide, and has a specific action on CD4 lymphocytes, where it inhibits calcineurin, an essential component of the IL-2 system (38). Both IL-2 expression and IL-2 surface receptors are inhibited, which depresses the CD4 lymphocyte’s ability to activate and to recruit, so that CD4-driven inflammation is reduced (38). In our case, GC therapy combined with CsA suppressed the increased cell populations of CD8^+^ T cells, B cells, NK cells as well as nonclassical monocytes. Therefore, we speculate that GC therapy combined with CsA may be a useful treatment for this case because of the similar pharmacological effects of both agents on VKH disease.

We had a good opportunity to examine the immune profile of not only the patient with panuveitis but also BNT162b2 vaccine-induced immune fluctuations in a healthy subject (**Table 2** and **Figure 2**). When infected with SARS-CoV-1 or Middle-east respiratory syndrome coronavirus similar to SARS-CoV-2 (39), innate immunity is augmented by activation of pattern recognition receptors (40). In this study, the sample from the healthy control after the third dose of BNT162b2 vaccine was collected approximately one month after the vaccination. In that sample, the immune cell populations of granulocytes (predominantly neutrophils and basophils), monocytes, and mDCs that are APCs were increased. Therefore, innate immunity may be predominantly activated approximately one month after the third vaccination. On the other hand, the activation of acquired immunity primarily involving B cells and T cells is speculated to occur in the early phase after COVID-19 vaccination, when the increases of SARS-CoV-2 spike 1-specific immunoglobulin G and neutralizing antibodies are induced (41). Therefore, the changes in immune profiles of the healthy control before and after BNT162b2 vaccination may provide a valuable source of information for understanding immune responses following BNT162b2 vaccination.

CRS developed in severe COVID-19 patients with ARDS (5, 6) is characterized by elevation of plasma TNFα and IL-6 levels (40). Our patient did not manifest symptoms of CRS, but the B cell and CD8^+^ T cell populations were increased at the time of onset. On the other hand, HLA-DR expression, B cells and T cells are decreased in COVID-19 patients with ARDS (7). Therefore, we could presume that BNT162b2 vaccine acted effectively as an immune activator for COVID-19 in our patient. SARS-CoV-2 can disturb self-tolerance and trigger autoimmune responses through immunological cross-reactivity with host cells (42). VKH disease is a T-cell-mediated autoimmune disease against one or more antigenic components of melanocytes (30, 43, 44). Assuming that BNT162b2 vaccine-induced COVID-19 mimicry occurred in this case, we speculate that the panuveitis resembling VKH disease may be caused accidentally by multiple factors including HLA-DR4 positivity as a genetic risk factor and immunological cross-reactivity to melanocyte epitopes. So, the possibility for screening tests of genetic predisposition to develop autoimmune diseases before vaccine administration is presumed, and in future, larger case series studies are required to elucidate it.

This study has several limitations (1). As this study is a case report, the immune profiles of the patient with BNT162b2 vaccine-related panuveitis and the healthy control cannot be generalized. In the future, larger case series studies are expected to reveal the immune profiles of COVID-19 vaccinated individuals that could be an aid to assess the efficacy and safety of the vaccination, and to predict the onset of potential side effects (2). The healthy individual was used as a relative control because he was not a VKH patient (3). Direct comparison of the immune profiles between the patient at the time of onset and the healthy control after the third dose of BNT162b2 vaccine is not appropriate, because the number of vaccine doses received and the number of days after vaccination differed (4). Real-time reverse transcription polymerase chain reaction (RT-PCR) testing for SARS-CoV-2 RNA using nasopharyngeal swab was not performed in this patient. At that time, administrative screening for SARS-CoV-2 by RT-PCR testing in Japan could only be performed for patients strongly suspected of COVID-19, who had fever of 37.5°C or higher, respiratory symptoms, and suspected pneumonia requiring hospitalization. In our patient, COVID-19 was not suspected from the systemic symptoms on admission, and thereafter no fever and respiratory symptoms were observed during GC therapy.

## Data availability statement

The original contributions presented in the study are included in the article/**Supplementary Material**. Further inquiries can be directed to the corresponding author.

## Ethics statement

Ethical review and approval was not required for the study on human participants in accordance with the local legislation and institutional requirements. The patients/participants provided their written informed consent to participate in this study.

## Author contributions

TS and MT drafted the manuscript. TS and MT reviewed and edited the manuscript. TS and YN performed cytokine measurements. TS and YN performed phenotyping of peripheral blood mononuclear cells. RN involved in treatment of this case, and collected the data. DS provided peripheral blood samples as a healthy control. All authors contributed to the article and approved the submitted version.

## Funding

TS received a Grant-in-Aid for Advanced Medical Development from National Defense Medical College, and Research Grant from Daiwa Securities Health Foundation. MT is supported by a Grant-in-Aid for Scientific Research C from the Japan Society for the Promotion of Science (16K11337).

## Conflict of interest

The authors declare that the research was conducted in the absence of any commercial or financial relationships that could be construed as a potential conflict of interest.

## Publisher’s note

All claims expressed in this article are solely those of the authors and do not necessarily represent those of their affiliated organizations, or those of the publisher, the editors and the reviewers. Any product that may be evaluated in this article, or claim that may be made by its manufacturer, is not guaranteed or endorsed by the publisher.
