# Supplementary material for: Case report: Bilateral panuveitis resembling Vogt-Koyanagi-Harada disease after second dose of BNT162b2 mRNA COVID-19 vaccine
Source: Front Immunol. 2022 Sep 29;13:967972. doi: 10.3389/fimmu.2022.967972 (PMC9556971; doi:10.3389/fimmu.2022.967972)
Supplement: Supplementary file 1 [file DataSheet_1.docx]

**
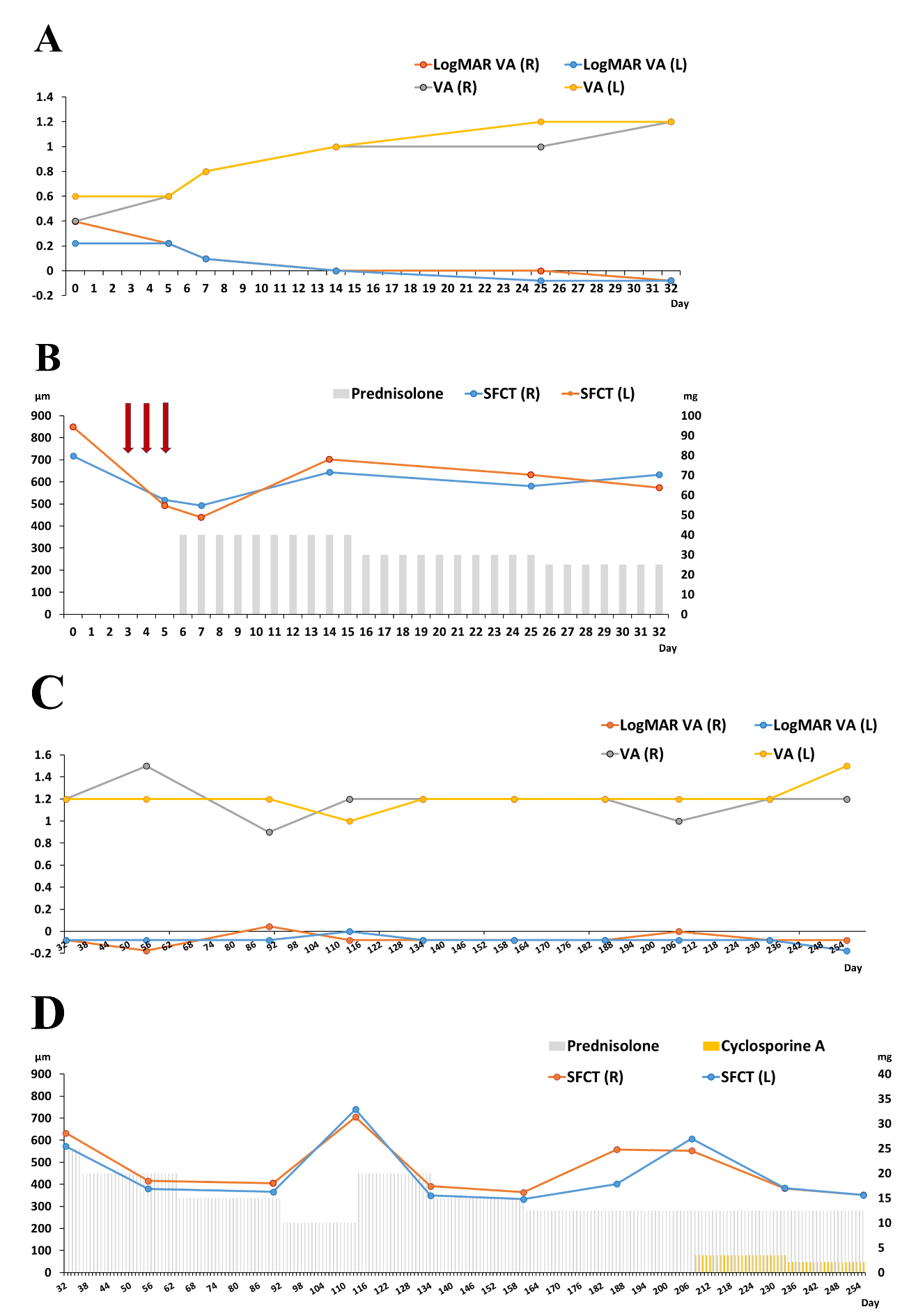
**

**Supplementary Figure 1** | Clinical course of the patient with panuveitis resembling VKH disease after admission. Time courses of **(A)** VA, **(B)** SFCT and oral dose of prednisolone in the acute phase, and timelines of **(C)** VA, **(D)** SFCT and oral doses of prednisolone and cyclosporine A in the inactive phase are shown. Left vertical axis shows SFCT, and right vertical axis means dose of prednisolone and cyclosporine A in **(B, D)**. Red arrows in **(B)** indicate intravenous pulses of methylprednisolone (1,000 mg/day for 3 days). Dosage units of prednisolone and cyclosporine A are mg/day and mg/kg/day, respectively. VA; best corrected visual acuity on the decimal chart, logMAR VA; VA converted to logMAR, SFCT; subfoveal choroidal thickness, VKH; Vogt-Koyanagi-Harada.

**
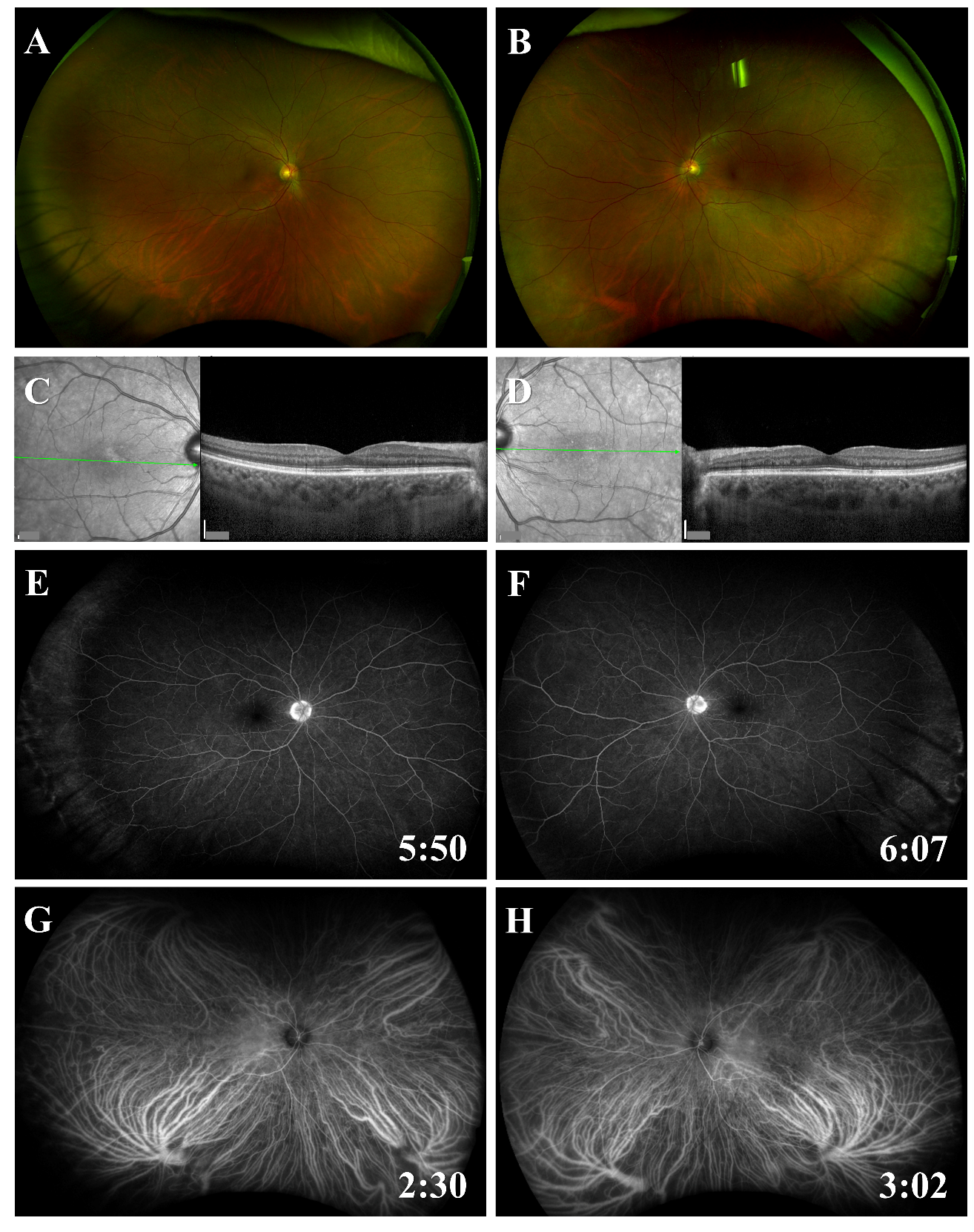
**

**Supplementary Figure 2** | Fundus findings of panuveitis in the inactive phase. Color fundus photographs taken approximately seven months later show disappearance of SRDs in the posterior retina, as well as redness and swelling of the optic disc in **(A)** the right eye and **(B)** the left eye. EDI-OCT images reveal almost normal structures of retinal layers without SRDs in **(C)** the right eye and **(D)** the left eye. FA images indicate disappearance of SRDs, and persisting hyperfluorescence in the optic disc in **(E)** the right eye and **(F)** the left eye. IA images present disappearance of dark patches in **(G)** the right eye and **(H)** the left eye. The time of photography after administration of FA or IA is indicated in the lower right corner. Scale bars (white vertical bar) in **(C, D)**: 200 μm. EDI-OCT; enhanced depth imaging optical coherence tomography, FA; fluorescein angiography, IA; indocyanine green angiography, SRD; serous retinal detachment.

**Supplementary Table 1** | Hematologic data of the patient with panuveitis resembling VKH disease at the time of onset.

IgG and IgM levels of HSV, VZV, CMV, EB and mumps were determined by EIA. ACE; angiotensin converting enzyme, ALT; alanine aminotransferase, APC; activated protein C, APTT; activated partial thromboplastin time, AST; aspartate transaminase, BUN; blood urea nitrogen, Ca; calcium CK; creatine kinase Cl; chlorine, CMV; cytomegalovirus, CRP; C-reactive protein, EB; Epstein-Barr virus, EIA; enzyme immunoassay, ESR; erythrocyte sedimentation rate, FBS; fasting blood sugar, FDP; fibrin/fibrinogen degradation products, Hb; hemoglobin, hBNP; human brain natriuretic peptide, HBsAg; hepatitis B surface antigen, HCV; hepatitis C virus, Ht; hematocrit, HSV; herpes simplex virus, Ig; immunoglobulin, IP; inorganic phosphorus K; potassium, LDH; lactate dehydrogenase, Na; sodium, MCH; mean corpuscular hemoglobin, MCHC; mean corpuscular hemoglobin concentration, MCV; mean corpuscular volume, PT; prothrombin time, PT-INR; prothrombin time-international normalized ratio, RPR; rapid plasma reagin, RBC; red blood cell, RDW; red blood cell distribution width, sIL-2R; soluble interleukin-2 receptor, TPHA; treponema pallidum hemagglutination test, VKH; Vogt-Koyanagi-Harada, VZV; varicella-zoster virus, WBC; white blood cells, 1 h; 1 hour.

**Supplementary Table 2** | Cerebrospinal fluid data of the patient at the time of onset.

CSF; cerebrospinal fluid, CFP; cerebrospinal fluid pressure.

**Supplementary Table 3** | Hematologic data of the patient in the inactive phase.
